# A Clinico-Pathological Study and Evaluation of Factors Associated With Poor Outcomes in Patients With Necrotizing Fasciitis in a Tertiary Care Hospital at Eastern India

**DOI:** 10.7759/cureus.112351

**Published:** 2026-07-09

**Authors:** Amresh Kumar, Rahul Saxena

**Affiliations:** 1 Department of General Surgery, Manipal Tata Medical College, Jamshedpur, IND; 2 Department of General Surgery, Tata Main Hospital, Jamshedpur, IND

**Keywords:** amputation, diabetes mellitus, mortality, soft tissue infection, surgical debridement

## Abstract

Introduction: Necrotizing fasciitis (NF) is a rapidly progressive, life-threatening soft tissue infection characterized by necrosis of the fascia and subcutaneous tissues, with high rates of amputation and mortality. Early diagnosis and aggressive surgical intervention are critical determinants of outcome, yet data from eastern India remain scarce. This study aimed to describe the clinicopathological profile of NF and evaluate factors associated with poor outcomes in patients admitted to a tertiary care center in Jharkhand.

Methods: This hospital-based observational study was conducted in the Department of General Surgery, Tata Main Hospital, Jamshedpur, from November 2020 to March 2022. One hundred consecutive patients diagnosed with NF were enrolled. Demographic data, clinical features, microbiological findings, and surgical management were recorded using a predesigned proforma. In-hospital mortality was used as the surrogate for poor outcomes. The chi-square test was used to assess associations between clinical variables and mortality, with p<0.05 considered statistically significant.

Results: The mean age of participants was in the 41-60-year group, with a male preponderance (76%). Diabetes mellitus was the most common comorbidity (66%). The lower extremity was the most frequently affected site (75%). *Staphylococcus* species (29%) and *Escherichia coli* (25%) were the predominant isolates. Debridement was performed in 97% of patients, and amputation was required in 18%. In-hospital mortality was 14%. On statistical analysis, diabetes mellitus (p=0.022), delayed or absent debridement (p=0.008), requirement of multiple surgeries (p=0.002), and amputation (p=0.009) were significantly associated with poor outcome.

Conclusion: Diabetes mellitus, delayed or absent debridement, requirement of multiple operative sittings, and amputation were significantly associated with in-hospital mortality in NF. Early clinical recognition and prompt aggressive surgical intervention, with meticulous management of diabetes, are essential to improve outcomes in this life-threatening condition.

## Introduction

Necrotizing fasciitis (NF) is a rapidly progressive, life-threatening soft tissue infection characterized by necrosis of the fascia and subcutaneous tissues, with relative sparing of the underlying muscle in early stages [1]. Though uncommon, its incidence ranges from 0.3 to 15 cases per 100,000 individuals, with amputation rates of 20-50% and mortality rates reaching 30-40%, making it one of the most feared surgical emergencies encountered in clinical practice [2].

The condition is notoriously difficult to diagnose in its early stages, as initial clinical manifestations often mimic cellulitis or abscess. This diagnostic ambiguity leads to delays in surgical intervention, which are strongly correlated with adverse outcomes [3]. Poor prognosis in NF has been linked to advanced age, uncontrolled diabetes, immunosuppression, and delayed surgery. Diabetes mellitus, in particular, stands out as the most prevalent comorbidity, predisposing patients to polymicrobial infections, higher rates of amputation, and increased perioperative morbidity [4].

The cornerstone of management remains early and aggressive surgical debridement, complemented by broad-spectrum antibiotics and intensive supportive care. Despite growing awareness, NF-related mortality has shown an upward trend since 2014, attributed to the rising prevalence of diabetes, obesity, and alcohol use disorders [5]. Recent data from the Indian subcontinent remains sparse, and existing studies indicate that the microbial profile and clinical determinants of outcome in this region may differ significantly from Western cohorts [6-8].

Given the burden of NF in resource-limited settings and the paucity of regional data from Eastern India, there is an unmet need for studies that describe the clinicopathological profile and systematically evaluate factors associated with poor outcomes in this patient population. The present study was therefore undertaken to address this gap among patients presenting to the Department of Surgery at a tertiary care center in Jamshedpur, Jharkhand.

## Materials and methods

The present hospital-based observational study was conducted in the Department of General Surgery, in a tertiary care center (Tata Main Hospital, Jamshedpur), in eastern India from November 2020 to March 2022, following approval from the Institutional Ethics Committee of the study hospital (vide reference number TMH/AC/IEC/NOV/035/2020 dated 23/11/2020). The study has been reported in line with the STROBE guidelines for observational studies.

Study participants and sample size

All consecutive patients admitted to the Department of General Surgery with a clinical and operative diagnosis of NF and who provided written informed consent were included. Patients who declined consent or had incomplete records were excluded.

The sample size was calculated using the standard formula for prevalence studies. Based on departmental audit data, the in-hospital prevalence of NF was estimated at 6.9%. Assuming a 95% confidence level (Z = 1.96) and 5% absolute error (d = 0.05), the minimum calculated sample size was 99, rounded up to 100 for convenience.

Study procedure

A predesigned, pretested case record form was used to collect relevant information at the time of admission and during the inpatient period. Demographic data including age, sex, and relevant medical history were recorded. All patients underwent a thorough clinical examination, and the site and extent of infection, clinical features at presentation, and intraoperative findings were documented. Tissue fluid collected intraoperatively was sent for aerobic and anaerobic bacterial culture and sensitivity. Details of surgical management, including time of debridement, number of operative sittings, and amputation, were recorded. The primary outcome of interest was in-hospital mortality, which was used as the surrogate for poor outcomes.

Outcome measures

The primary outcome of interest was in-hospital mortality, which was used as the surrogate for poor outcomes.

Data entry and statistical analysis

Data were entered into Microsoft Excel 2016 (Microsoft Corporation, Redmond, USA) and analyzed using IBM SPSS Statistics for Windows, Version 22 (Released 2013; IBM Corp., Armonk, New York, United States). Categorical variables were expressed as frequencies and percentages, and continuous variables as mean ± standard deviation. Factors associated with poor outcome (mortality) were assessed using the chi-square test or Fisher's exact test (when the expected value in at least one cell is less than 5). For all analyses, a p-value of less than 0.05 was considered statistically significant.

## Results

A total of 100 patients diagnosed with N were included in the study. The majority of patients were in the 41-60-year age group (n=39, 39%), closely followed by the 61-80-year age group (n=34, 34%). Male patients accounted for 76% of cases (n=76). Diabetes mellitus was the most prevalent comorbidity, present in 66 (66%) patients (Table 1).

**Table 1:** Baseline characteristics of the study participants (N=100)

Characteristics	Frequency n (%)
Age	
≤40 years	15 (15.0)
41-60 years	39 (39.0)
61-80 years	34 (34.0)
>80 years	12 (12.0)
Gender	
Male	76 (76.0)
Female	24 (24.0)
Relevant medical history	
Diabetes mellitus	66 (66.0)
Chronic immunosuppressive medication	2 (2.0)
Peripheral vascular disease	24 (24.0)

The lower extremity was the most commonly affected site, involved in 75% of patients, with the entire lower limb affected in 36% and the foot in 26% of cases. The perineal region was the second most frequent site, with scrotal involvement in 20% and penile involvement in 2% of cases. Only 4% of cases involved the upper extremity (Table 2).

**Table 2:** Site of infection among the study participants (N=100) *Multiple sites may be affected in one participant

Site of infection*	Frequency n (%)
Lower extremity	
Entire lower limb	36 (36.0)
Knee	1 (1.0)
Leg	8 (8.0)
Foot	26 (26.0)
Toe	4 (4.0)
Upper extremity	
Entire upper limb	1 (1.0)
Hand	3 (3.0)
Perineal region	
Scrotum	20 (20.0)
Penis	2 (2.0)
Other parts	
Gluteal region	1 (1.0)
Anterior abdominal wall	2 (2.0)
Back	1 (1.0)

**Figure 1:**
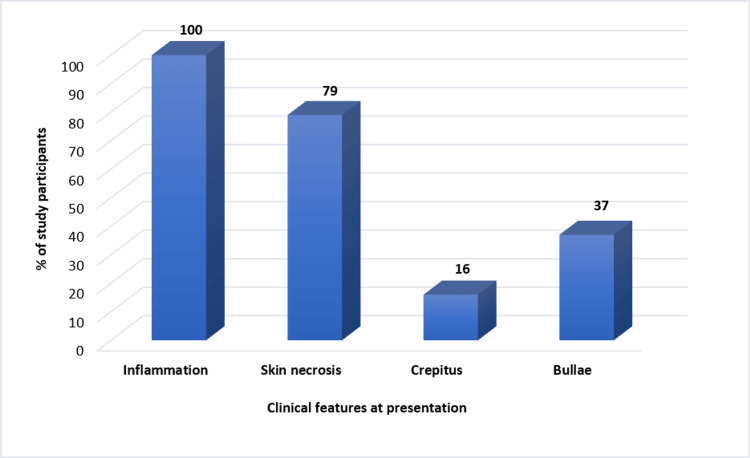
Clinical features at presentation among the study participants (N=100)

Inflammation was universally present at presentation (100%), while skin necrosis, bullae, and crepitus were observed in 79%, 37%, and 16% of cases, respectively (Figure 1).

On microbiological analysis, the most frequently isolated organism was *Staphylococcus* species (29%), followed by *Escherichia coli* (25%), *Klebsiella *(12%), and *Pseudomonas *(9%). Polymicrobial infection was documented in 11% of cases, while no bacterial growth was reported in 6% of samples (Table 3).

**Table 3:** Infecting organisms isolated from tissue fluid culture (N=100)

Tissue fluid culture report	Frequency n (%)
Staphylococcus	29 (29.0)
Enterococcus	4 (4.0)
Pseudomonas	9 (9.0)
Escherichia coli	25 (25.0)
Klebsiella	12 (12.0)
Proteus	1 (1.0)
Morganella morganii	3 (3.0)
Polymicrobial	11 (11.0)
No bacterial growth	6 (6.0)

The in-hospital mortality was recorded in 14 patients (14%). On analysis of factors associated with poor outcomes, diabetes mellitus was found to be significantly associated with mortality, being present in 92.9% of the deceased patients compared to 61.6% of survivors (p=0.022). Delayed or absent debridement, requirement of multiple surgeries, and amputation were also significantly associated with mortality (p=0.008, p=0.002, and p=0.009, respectively) (Table 4).

**Table 4:** Factors affecting poor outcomes in the study participants (N=100) # p-value was calculated using the chi-square test or Fisher's exact test (as applicable); * p<0.05 was considered statistically significant.

Parameters	Total	Dead (n=14) n (%)	Alive (n=86) n (%)	ꭓ^2^ value	p-value^#^
Age					
< 60 years	54 (54.0)	6 (42.9)	48 (55.8)	0.814	0.367
≥ 60 years	46 (46.0)	8 (57.1)	38 (44.2)		
Gender					
Male	76 (76.0)	12 (85.7)	64 (74.4)	0.842	0.359
Female	24 (24.0)	2 (14.3)	22 (25.6)		
Diabetes mellitus					
Yes	66 (66.0)	13 (92.9)	53 (61.6)	5.233	0.022*
No	34 (34.0)	1 (7.1)	33 (38.4)		
Debridement					
Early debridement	97 (97.0)	12 (85.7)	85 (98.8)	7.125	0.008*
Delayed/ absent debridement	3 (3.0)	2 (14.3)	1 (1.2)		
Surgery					
Single	95 (95.0)	11 (78.6)	84 (97.7)	9.250	0.002*
Multiple	5 (5.0)	3 (21.4)	2 (2.3)		
Amputation					
Yes	18 (18.0)	6 (42.9)	12 (13.9)	6.815	0.009*
No	82 (82.0)	8 (57.1)	74 (86.1)		

## Discussion

The present study describes the clinicopathological profile and factors predicting poor outcome in 100 patients of NF admitted to a tertiary care center in eastern India. The demographic profile in our study revealed a male preponderance (76%), consistent with published literature reporting a male-to-female ratio of approximately 3:1, which has been attributed to differences in occupational exposure to trauma and a higher prevalence of predisposing comorbidities among men [1]. The majority of our patients were in the middle-aged to elderly group (41-80 years, 73%), corroborating findings from other Indian series [7,8]. This distribution likely reflects the higher burden of metabolic comorbidities in this age group. Diabetes mellitus was the most prevalent comorbidity in our cohort, present in 66% of patients, which is consistent with reported rates of 31-66% in the literature and underscores the well-established role of poor glycaemia control in impairing immune function, promoting polymicrobial invasion, and delaying wound healing [7,9].

The lower extremity was the predominant site of infection (75%), in keeping with other studies from India and the Asia-Pacific region [7,10]. The perineal region, representing Fournier's gangrene, accounted for 22% of cases, which aligns with prior reports documenting the perineum as the second most common site, particularly in diabetic men [1]. Upper extremity involvement was uncommon (4%), consistent with published series [1]. Inflammation was present universally at admission, while skin necrosis (79%), bullae (37%), and crepitus (16%) were present in a significant proportion. The relatively low prevalence of crepitus in our series is in agreement with existing literature, which describes it as a late sign present in only 16-18% of cases, and its absence should never be used to exclude the diagnosis [11]. The clinical picture of NF is notoriously deceptive in its early stages, often masquerading as cellulitis, leading to diagnostic delays that are independently associated with worse outcomes [12,13].

Microbiologically, Staphylococcus species were the most common isolate (29%), followed by *Escherichia coli* (25%) and *Klebsiella *(12%). This mixed Gram-positive and Gram-negative profile reflects the polymicrobial nature of NF in our setting, though the dominant organisms vary by region and patient population. Polymicrobial infection was documented in 11% of cases, which is lower than the rates reported in Western cohorts (up to 87%), possibly reflecting differences in the microbiological environment, antibiotic usage patterns, and the prevalence of specific comorbidities [8,10]. The presence of Gram-negative organisms such as *Klebsiella*, *Pseudomonas*, and *Morganella morganii* in diabetic patients warrants particular attention, as these are associated with more aggressive disease and higher rates of treatment failure [9].

The overall in-hospital mortality in our study was 14%. Diabetes mellitus was observed to be a significant predictor of mortality (p=0.022). This is well-supported by the literature. Diabetic patients with NF have been shown to have higher rates of polymicrobial infection, more extensive tissue necrosis at presentation, and significantly greater odds of amputation and death [4,9]. The metabolic derangements of poorly controlled diabetes, primarily hyperglycemia-mediated neutrophil dysfunction, impaired opsonization, and microvascular insufficiency, create a hostile environment that accelerates fascial necrosis and systemic sepsis [14]. In our cohort, 92.9% of those who died had diabetes, compared to 61.6% of survivors, reinforcing diabetes as a critical determinant of outcome in this condition.

A notable finding was the significant association between delayed or inadequate debridement and mortality (p=0.008). Early and aggressive debridement remains the single most important determinant of survival, and delays in operative intervention have consistently been linked to higher mortality across multiple series [15,16]. Similarly, the need for multiple surgeries was significantly associated with death (p=0.002), suggesting that patients requiring repeat debridement represented a subset with more extensive or rapidly progressive disease, biologically more aggressive infections, or suboptimal initial source control, all of which independently worsen prognosis. The significant association of amputation with mortality (p=0.009) in our study echoes findings from DeGenova et al., who demonstrated that diabetic NF patients were nearly three times more likely to require amputation than their non-diabetic counterparts and that the overall mortality for NF of an extremity was 26.1% [4]. Amputation in this context is both a marker of disease severity and a consequence of delayed or insufficient debridement, and its association with mortality likely reflects the advanced stage of infection at the time of intervention.

Contrary to some published series, age and gender were not significantly associated with mortality in our study. This may be attributable to the relatively small sample size limiting statistical power, or to the dominant confounding effect of diabetes masking the independent contributions of these variables. Larger multivariate analyses would be required to definitively establish or refute their independent roles.

The rising global burden of NF, driven by increasing prevalence of diabetes, obesity, and immune-compromising conditions, makes our study particularly relevant. Khan et al. demonstrated a 120.6% increase in NF-related mortality in the United States between 2003 and 2020, attributing this trend to the growing prevalence of metabolic risk factors and healthcare access disparities [5]. In the Indian context, where diabetes prevalence is among the highest in the world and healthcare resources are unevenly distributed, proactive identification of high-risk patients and early surgical intervention are essential strategies to reduce preventable deaths.

The present study has some limitations. First, it was conducted at a single tertiary care center, which may limit the generalizability of the findings to other settings. Second, although the calculated sample size was achieved, the relatively small cohort may have reduced the statistical power to detect certain associations. Third, only univariate analyses were performed; therefore, independent predictors of mortality could not be established through multivariable adjustment for potential confounders. Finally, poor outcome was defined solely as in-hospital mortality, and other clinically relevant outcomes such as long-term functional status, postoperative complications, quality of life, and readmissions were not evaluated. Future multicenter studies with larger sample sizes and multivariable analyses incorporating longer-term outcomes are warranted.

## Conclusions

Based on the findings of our study, it can be concluded that NF predominantly affects middle-aged to elderly men with diabetes mellitus, with the lower extremity being the most common site of infection. Diabetes mellitus, delayed or absent debridement, requirement of multiple surgeries, and amputation were significantly associated with in-hospital mortality. Early clinical recognition, prompt and aggressive surgical debridement, and meticulous management of diabetes are the cornerstones of improving survival in this life-threatening condition. Given the paucity of data from eastern India, this study adds important regional evidence and underscores the need for structured, multidisciplinary protocols for the management of NF in resource-limited settings.
